# Subjetividad farmacéutica en tiempos de crisis en Madrid: Entre la supervivencia, la cronificación y el “Debo ser yo”

**DOI:** 10.18294/sc.2025.5563

**Published:** 2025-03-06

**Authors:** María Reneses

**Affiliations:** 1 Doctora en Antropología. Investigadora posdoctoral. Instituto de Investigación Tecnológica, Escuela Técnica Superior de Ingeniería (ICAI), Facultad de Ciencias Humanas y Sociales, Universidad Pontificia Comillas, Madrid, España. mariareneses@gmail.com Universidad Pontificia de Comillas Escuela Técnica Superior de Ingeniería Facultad de Ciencias Humanas y Sociales Universidad Pontificia Comillas Madrid Spain mariareneses@gmail.com

**Keywords:** Salud Mental, Psicofármacos, Efectos Metabólicos Secundarios de Drogas y Sustancias, España

## Abstract

En los últimos años, la salud mental ha cobrado una enorme relevancia con una progresiva desestigmatización. Esto hace que cada vez más personas entiendan y analicen su sufrimiento en términos psicológicos. Pero para muchas personas que acceden a los dispositivos públicos de salud mental en una ciudad como Madrid, la principal opción de tratamiento es la medicación. Con el objetivo de analizar qué tipo de subjetividad se produce a partir de esa forma de intervención, se analizan los resultados obtenidos en una etnografía realizada entre 2012 y 2014 que incluyó la observación en las consultas de un centro de salud mental, 19 entrevistas en profundidad con consumidores de psicofármacos y un grupo de reflexión con encuentros periódicos. Entre los principales hallazgos encontramos la ambivalencia respecto a los fármacos y la necesidad de ajustes continuos para minimizar los efectos secundarios. En la subjetividad resultante se vuelven centrales aspectos como el miedo a la recaída y a los efectos secundarios; la autonomía, que choca con la idea de no intentarlo por uno mismo; la responsabilización, el sentimiento de vulnerabilidad y el autogobierno, aspectos claves de la subjetividad neoliberal.

## INTRODUCCIÓN

La cultura que tenemos en torno a la salud mental, la forma en que percibimos el padecimiento psíquico y su abordaje depende de nuestro contexto sociohistórico y, por tanto, no deja de cambiar^1^. No solo las formas de intervenir, las propias percepciones de la sociedad han cambiado desde la época del encierro a las reformas psiquiátricas. De acuerdo con la antropología médica y la antropología del sufrimiento social, nuestra relación con el sufrimiento y nuestras respuestas a él, y nuestra valoración sobre la salud mental y la atención a la salud mental se modifican, además, de acuerdo con las nuevas formas de gobierno. Cuando las categorías y las prácticas del sentido común cotidiano son sustituidas por la racionalidad biomédica se transforman los valores y las formas principales de subjetividad contemporánea^2^. 

En primer lugar, aunque cierto estigma permanece, sobre todo hacia grupos vulnerables, se ha producido una fuerte desestigmatización de los problemas de salud mental^3^. A pesar de que esta tendencia es anterior a la pandemia, se habría profundizado con posterioridad a esta^4^. Junto a la desestigmatización, la sociedad neoliberal cada vez invita más al manejo, la modulación y la mejora de las capacidades vitales de los seres humanos, y a la búsqueda de la optimización de la mente y de las emociones de las personas^5^. Junto a libros de autoayuda, *coaches* y psicólogos, prolifera el consumo de fármacos recetados por los médicos de atención primaria y los psiquiatras, como otra herramienta para conseguir la mejora del bienestar emocional.

Las personas son entonces cada vez más propensas a definir el sufrimiento psíquico o el malestar como un problema de salud mental y son también más propensas a buscar ayuda profesional para el mismo. En este contexto, resulta relevante analizar cómo afecta y modula la subjetividad de las personas la principal forma de intervención que tiene lugar en España, al menos desde el dispositivo público: la medicación psiquiátrica. Para ello se expondrá una parte del material empírico recogido para la tesis doctoral de la autora de este artículo^6^.

Pese a que podemos valorar la desestigmatización de la salud mental y la progresiva petición del aumento de recursos como algo positivo, es importante analizar cómo afecta esta centralidad de la salud mental a las personas que apenas pueden acceder a recursos terapéuticos. En el presente estudio, se analizan las características del consumo de fármacos y la subjetividad farmacéutica resultante, rescatando la voz y la experiencia de un grupo de consumidores que no ha sido tan estudiado como los diagnosticados con enfermedades graves: pacientes con diagnóstico más leve y con un padecimiento en gran parte reactivo a la situación de crisis y precariedad.

El contexto en el que se realizó el estudio fue el de la anterior crisis económica (2012-2014) en la que el desempleo y los desalojos eran habituales en todo el territorio español. La deficiencia de los mecanismos de cobertura social implicó que un alto número de la población pasara a vivir por debajo del umbral de la pobreza^7^, de forma especial en Puente de Vallecas, uno de los barrios más golpeados por la crisis de la región de Madrid^8^. Se trataba entonces (y ahora) de uno de los distritos de Madrid con menor renta per cápita, donde más se incrementó la desocupación y el precio de la vivienda, y donde se conjugó la peor percepción del estado de salud, peor esperanza de vida y peor nivel de renta de sus habitantes. El barrio es, además, uno de los de mayor dependencia de la asistencia pública, sin apenas cobertura privada o mixta^9^. El centro de salud mental donde se realizó la etnografía no solo estaba saturado por la demanda creciente y el recorte presupuestario. Existía, además, una amenaza de privatización que no llegó a tener lugar, pero que supuso que los contratos de los profesionales pasaron de ser de seis meses al comenzar el estudio, a serlo primero de tres, y después de un mes.

### Consumo de psicofármacos y determinación social

Aunque el consumo de psicofármacos en España ha sufrido un leve descenso en los últimos años^10^, probablemente debido a la relativa mejora económica, según las Naciones Unidas, en 2022^11^ España se encontraba entre los países con mayor consumo de psicotrópicos en el mundo, a la cabeza en el consumo de benzodiacepinas. Además, en un estudio realizado en 2023 se mostró que la mayor parte de los consumidores de estos medicamentos en la población asalariada española atribuyeron el consumo a motivos relacionados con el trabajo (un 11% así lo afirmó frente a un 3,6% que no)^12^. En los últimos años en España, los estudios muestran de forma sostenida los efectos de las condiciones socioeconómicas en la salud mental. El consumo de ansiolíticos y antidepresivos correlaciona de forma positiva con ser de clase baja, estar desempleado, tener problemas de vivienda o estar a cargo del cuidado de una persona discapacitada^13^^,^^14^^,^^15^. Respecto al género, las mujeres no solo siguen reportando peor salud mental, sino que se da junto a un sobrediagnóstico y medicalización que incluye una mayor prescripción de psicofármacos^16^.

No obstante, las cifras sobre consumo de psicofármacos son complejas, y no se pueden atribuir directamente a razones epidemiológicas. Como señala Lakoff^17^, no se trata ya de contaminación de la ciencia pura sino de una estructura de conocimiento interesado que liga el marketing a la ciencia en los niveles más profundos de la economía biomédica. El aumento en el consumo de antidepresivos puede indicar un aumento en la prevalencia de depresión, o una estrategia de marketing para que estos sean indicados en los casos de ansiedad en lugar de las benzodiacepinas^18^.

Además, el exceso de prescripción, así como los fármacos que son prescritos, no solo tienen que ver con la industria farmacéutica y con el biologicismo que caracteriza la psiquiatría contemporánea. También es una forma de dar respuesta a una demanda ante la ausencia de otras alternativas. La prescripción es un acto social por el que el médico reconoce el padecimiento del paciente y le muestra su voluntad de ayudarle. Los medicamentos se ven como la esencia de la práctica médica y, por ello, la prescripción es lo que se espera de los médicos^19^. Si partimos de que la población mundial cada vez está más preocupada por la salud mental, no se ha prestado suficiente atención al conocimiento sociocultural, tanto de las enfermedades como del efecto de su tratamiento^20^.

### Subjetividad farmacéutica

Los fármacos no solo tienen un efecto químico, también actúan a través del imaginario que se construye en torno a ellos. Son tanto producto como productores de la cultura humana. Como facilitadores del autocuidado dirigen los pensamientos y las acciones de las personas, influyendo en la vida social, en lo que podemos denominar la producción de una subjetividad farmacéutica.

Esta subjetividad está formada por el *self* y el imaginario farmacéutico, que actúan como sus dos caras. Jenkins^21^ define el *self* (sí mismo) como la suma de los procesos por los que el sujeto se orienta en el mundo y con los demás. El otro elemento sería el imaginario, es decir, la dimensión de la cultura que determina las posibilidades concebibles de la vida humana. El imaginario, que no opera solo en los consumidores, puede englobar desde sentimientos y fantasías a valores e ideales, de modo que el marketing de un fármaco o una enfermedad produce un imaginario que incluye unas determinadas conductas o sentires como adecuados y otras como sancionables y no deseables.

Para Pazos^22^, la constitución de la subjetividad incluye procesos de incorporación entendidos como formas de hacer y formas de decir, como sistemas de disposiciones o *habitus* con los que se articula la experiencia de sí. Las categorías aplicadas a la clasificación de los individuos terminan produciendo determinado tipo de personas que no existían con anterioridad^23^. 

Nikolas Rose^24^ describe el fenómeno de la progresiva farmacologización y su reflejo en la subjetividad como *self* neuroquímico. Para el autor, supone un cambio de la comprensión psicológica del individuo a la individualidad somática, es decir, un individuo que cada vez se piensa más en términos biomédicos y que trata de modificarse, curarse o mejorarse actuando sobre el cuerpo. Para Emily Martin^25^ la persona farmacéutica incorpora tanto las estrategias de marketing de las empresas farmacéuticas como las ambivalencias que manejan los consumidores debido a los efectos secundarios.

Por último, Biehl y Morant-Thomas^26^ recurren a la concepción deleuziana de la subjetividad para explicarla no solo como producto sino también como productora de nuevos universos sociales. Se trata de una noción que permite superar el debate entre la agencia y la estructura al considerarse ambas como afectivamente interconectadas, en lugar de como excluyentes. Según estos autores la subjetividad tiene más de devenir que de estructural. La intervención biomédica produce subjetivación en cuanto que implica nuevas ideas sobre lo que son las personas, lo que deberían ser y lo que pueden esperar ser.

Una de las áreas que más se ha investigado con relación a la subjetividad producida en el encuentro médico ha sido la de las enfermedades y las intervenciones dirigidas a las mujeres y su relación con la conformación de los roles de género, por ejemplo, a través de los fármacos destinados a aliviar los dolores menstruales^27^, o de la reproducción asistida^28^. También han sido estudiados los efectos en la subjetividad y la forma de percibir el cuerpo de los tratamientos crónicos como los retrovirales de VIH^29^ o los fármacos para la diabetes^30^.

Las investigaciones sobre la experiencia del consumo de psicofármacos en pacientes con diagnósticos graves revelan algunas características como la imposibilidad de aislar los efectos secundarios no deseados de la totalidad de la experiencia de la medicación, y la importancia de los efectos situados en el contexto de la vida de las personas^31^. También señalan que existen barreras institucionales que limitan el derecho a abandonar el tratamiento, algo que se suele realizar de forma autónoma utilizando estrategias de autoayuda y de apoyo social, sobre todo para lidiar con el “efecto de resaca”^32^. 

A continuación, describiremos la experiencia con la medicación psiquiátrica de un grupo de personas caracterizado por tener un diagnóstico leve y, en su mayoría, considerado reactivo a la situación de crisis económica.

## METODOLOGÍA

Entre el año 2012 y 2014 realicé trabajo de campo en tres fases para mi tesis doctoral con personas en seguimiento en el centro público de salud mental del barrio Puente de Vallecas. Tras una primera fase de observación a diario durante dos meses en la consulta de una psiquiatra y una psicóloga, en una segunda fase conduje 19 entrevistas individuales con consumidores de psicofármacos. La tercera y última fase consistió en un grupo formado por algunas de las personas entrevistadas que se reunió de forma periódica a lo largo de dos años. A pesar de que los resultados se centran en la segunda y la tercera fase, se refleja el total de la etnografía, ya que el trabajo en tres momentos posibilitó llevar a cabo tres tareas diferenciadas: la primera fase de *observación* estuvo destinada a la apertura de la mirada y a la delimitación del objeto de estudio; la segunda de *entrevistas* tuvo como finalidad la concreción y elaboración de hipótesis; la tercera y última de *formato grupal* se dedicó a la contrastación de esas hipótesis. 

La observación posibilita contrastar lo que aparece en el encuentro clínico y lo que se expresa fuera de él. Según Kleinman^33^, para entender los cambios subjetivos que tienen lugar en determinados contextos históricos, no basta con atender a las transformaciones en la macropolítica, hay que analizar también a los mundos locales en los que aparecen esas representaciones culturales y esos procesos colectivos. La etnografía, en concreto, es una técnica que se utiliza para ese estudio, también en el contexto médico^21^^,^^34^^,^^35^^,^^36^^,^^37^. La etnografía permite estudiar los patrones de la formación del *self*, la forma en que la vida interior y los valores están cambiando, y cómo estas transformaciones afectan al sufrimiento y a nuestro modo de interpretarlo^26^. Este análisis también incluye la comprensión del significado de la salud mental y de la asistencia psiquiátrica en este contexto.

Las personas entrevistadas fueron seleccionadas a partir de lo encontrado en la observación. La mayoría de las personas que se vieron en la consulta identificaron el comienzo de su malestar con algún acontecimiento o situación reciente, como el sufrimiento derivado del trabajo asalariado y del desempleo y del trabajo reproductivo no remunerado, especialmente el cuidado a tiempo completo de personas dependientes. Por este motivo, casi todas las personas participantes seleccionadas tenían como principal problemática una de estas cuestiones, aunque también se incluyó a un participante sin claro precipitante social (Tabla 1), pero que en la consulta pareció interesado por los efectos de la medicación. 

**Tabla 1:** Características de las personas entrevistadas. Madrid, 2012-2014.

N°.	Nombre ficticio	Género	Edad	Demanda/problema principal	Situación socio-laboral
1	Rocío	Mujer	49	Distimia	Limpiadora desempleada con fibromialgia e hijo con discapacidad
2	Concepción	Mujer	55	Cuidados	Cuidadora con problemas económicos (depende de su pareja con la que tiene problemas)
3	Diana	Mujer	28	Estrés laboral	Teleoperadora
4	Matilde	Mujer	60	Cuidados	Cuidadora con problemas económicos
5	Beatriz	Mujer	35	Problemas de pareja y laborales	Dependienta
6	Eduardo	Hombre	26	Despido	Dependiente
7	Carmen	Mujer	40	Problemas (“acoso”) laboral	Limpiadora
8	Clara	Mujer	26	Estrés laboral	Alterna contratos precarios con desempleo en investigación
9	Pedro	Hombre	58	Despido tras lucha sindical	Desempleado exsindicalista
10	Lucas	Hombre	47	Estrés laboral	Cocinero
11	Blanca	Mujer	49	Estrés laboral	Limpiadora. Marido e hija desempleados.
12	Hans	Hombre	45	Ansiedad	Sin situación reactiva
13	Marta	Mujer	21	Alterna trabajo precario con desempleo	No se puede independizar, tiene que aportar dinero en casa
14	Inmaculada	Mujer	31	Desempleo	Deudas, cáncer en tratamiento
15	Carlos	Hombre	47	Problemas económicos	Trabaja, pero no llega a fin de mes
16	Isabel	Mujer	50	Problemas laborales	Marido desempleado, excuidadora
17	Lola	Mujer	55	Problemas laborales	Derivados de un accidente laboral
18	Patricia	Mujer	38	Acoso laboral	No tiene problemas económicos
19	Olga	Mujer	50	Desempleo	Ella y su marido están desempleados, dependen de pensión de padres.

Fuente: Elaboración propia.

Se realizaron 19 entrevistas en profundidad. La selección vino dada, por un lado, por cierta representatividad, o lo que Guber^38^ denomina la redundancia de la vida social. En este caso, los cuidados, los problemas laborales y el desempleo aparecieron como temas que por lo reiterados se mostraron como manifestaciones dominantes dentro de la práctica de intervención. Por otro lado, la etnografía, a diferencia de otras metodologías, no se limita a una labor de representación. Como explica Han^39^, la singularidad puede ser igualmente interesante, por lo que la selección de los pacientes estuvo marcada también, en algunos casos, sin desencadenante claro, por una perspectiva de estudio de caso, más allá de la probabilística. 

Todas las entrevistas se realizaron fuera del centro de salud para que los pacientes separaran los contextos y que no respondieran pensando que podía condicionar su tratamiento o pensando en la respuesta más “científica”. Fue necesario marcar un rol diferenciado frente a dicho contexto e insistir en que no había respuestas buenas o malas, y que la entrevista trataba de recoger, precisamente, los elementos que con frecuencia quedan fuera del encuentro médico. En el caso de las cuidadoras -en su mayoría mujeres- solo se pudo concertar una entrevista con dos mujeres y en su domicilio.

Se realizó una entrevista abierta, de tipo etnográfico, dirigida en parte por el entrevistado, pero con unos elementos fijos a abordar. Al tratarse de una entrevista sobre cuestiones íntimas, a menudo difíciles de verbalizar, el entrevistador debe mantener una escucha activa y sensible, prestando atención a la variación entre lo explícito y lo implícito en el discurso^40^.

El grupo de apoyo mutuo y reflexión, además de servir como fuente de material empírico, se incluyó para que fuera de utilidad a los pacientes y para incorporar a la investigación una parte final, más propositiva, que describiera algunas alternativas viables a la intervención biomédica del malestar. De este modo, se desarrolló una suerte de antropología clínica^35^ o como locus de resistencia^41^. Aunque el grupo se les ofreció a todos los participantes, cuatro no participaron porque estaban mejor, tres por falta de disponibilidad y uno por vergüenza. El grupo, que se reunió durante dos años, además de una finalidad empírica tuvo sobre todo una dimensión ética de devolver a las personas participantes parte del conocimiento que habían generado. Fue también una forma de contribuir con la construcción de alternativas de corte más comunitario, y una demanda que salió en varias entrevistas, como la de Rocío: “*A no ser que alguien tenga un poquito de amor propio y diga por su cuenta ‘voy a hacer un grupo con esa gente, pero claro, esa gente necesita dinero’*” (Rocío, M49).

La observación se registró mediante el diario de campo, y los grupos se grabaron y transcribieron solo en fragmentos concretos. Las entrevistas se transcribieron de forma íntegra y se analizaron mediante un análisis de contenido categorial, es decir, se descompuso el texto en unidades que se agruparon en categorías según áreas temáticas^42^. Las categorías encontradas fueron: efectos de fármacos, dificultad de precisar efectos, desafección, efecto *zombie*, cambios en la percepción del *self*, supervivencia/mejora, efectos secundarios, dificultades abandono, dependencia, contradicción dependencia/autonomía, autogestión, percepción, estigma y abuso en la prescripción. Dentro de esta estructura, se incluyeron citas directas para ilustrar las ideas más relevantes. Finalmente, a partir de los datos extraídos con el sistema de categorías, se realizó la conceptualización e interpretación. 

El comité de ética del Hospital General Universitario Gregorio Marañón aprobó el estudio bajo el código de aprobación 319/11-IP, y todas las personas participantes firmaron un consentimiento informado. Los nombres de los participantes son ficticios para garantizar su confidencialidad.

## RESULTADOS

“*Ahora estoy colocaó, estoy equilibrado. Pero es artificial, quiero acostumbrarme a no estar entre algodones*”. (Hans, H45)

“*Estoy perdiendo la confianza en mí misma... Al tomar la medicación siento que lo estoy dejando de intentar por mí misma*”. (Concepción, M55)

### La intervención como prescripción

Todas las personas que acudieron a la consulta llegaron con el tratamiento ansiolítico o antidepresivo pautado por atención primaria, tras un tiempo sin encontrar mejoría. La relación que establecen los pacientes con los fármacos desde el mismo comienzo del tratamiento es compleja y ambivalente, pero existe una postura mayoritaria que considera que se abusa de la prescripción:

“*Yo pienso que siempre te hace mejor el hablar, mejor que las pastillas, no soy amigo de la química. Siempre hablar con una persona y conectar* [...] *La terapia es lo mejor, si se pudiera evitar tomar pastillas con la terapia*”. (Eduardo, H26)

“*No es con una pastillita y se te quita todo. Y lo que tendría que haber más es más terapia. Menos pastilla y más terapia. Antes de llegar a estar tan mal, si te hubieran dado terapia no llegas tan mal para las pastillas, más de uno seguro que nos quitaríamos las pastillas, o no tendríamos que haber llegado a ellas*”. (Concepción, M55).

El abuso en la prescripción se plantea como el aumento de la dosis o como el añadir pastillas nuevas:

“*Cada día te dan una nueva, y dices voy a acabar tomando 50 pastillas al día*”. (Diana, M28)

“*Te dicen empiezas por 20, 40, 80, y si no te subo. Joder, no fastidies. Y yo he visto a mi padre con una depresión que ha estado en cama un año y pico, y medicado hasta las trancas, le dejaban allí frito*”. (Hans, H25)

En general, se critica que el tratamiento guíe la casi totalidad de la entrevista clínica:

“*Qué te pasa, uy, qué mal estás, qué mal estás, tómate esto, esto, y esto. Eh, ves que estoy llorando y ya sabes la pastilla que me tengo que tomar, vale*”. (Diana, M28)

“*Hola, buenas tardes, qué tal, venga toma, pun, pun, pun, tira de receta* […] *El psiquiatra te dice, ¿qué tal? ¿cómo estás? ¿cómo va la medicación? Sigue con la lucha y tómate esto*” (Carlos, H47)

### Los efectos: el pharmakon

-“*Y con un lorazepam, luego durante el día ¿no te duermes?*”. (Beatriz, M35)

-“*No*”. (Hans, H45)

-“*Jo, debo ser yo…*”. (Beatriz, M35)

Casi todos los pacientes reconocen efectos favorables en la medicación, entre los que destacan estar más tranquilo, dormir mejor y dar menos vueltas a las cosas. No obstante, hay una idea casi general y es que los fármacos son una “ayuda” pero no curan:

“*No es que me hagan estar feliz, pero sí estoy mejor*”. (Beatriz, M35)

“*Tampoco noto una gran mejoría*”. (Diana, M28)

“*Alegría no dan, pero serenan*”. (Pedro, H58)

“*Es como cuando tienes una jaqueca, ayuda, pero no te la va a quitar*”. (Carlos, H47)

Lucas, pese a ser uno de los menos críticos con los psicofármacos, expresa que los usa para “*poder funcionar, salir a la calle*”. Eduardo (H26), que describe cómo el efecto calmante de los fármacos le permite, aunque no llegue a salir, tener la cabeza lo suficientemente despejada para jugar a la consola o al ordenador. En palabras de Hans:

“*Con la medicación sí, sí parece que te notas mejor, pero tampoco de forma tremenda, simplemente para sobrevivir*”. (Hans, H45)

La sensación más euforizante se nota solo durante las primeras dos o tres semanas de consumo, y luego desaparece. El principal efecto que se describe en los antidepresivos es tener más ganas de hacer cosas, como un empuje a la acción, más que una sensación de felicidad, y en los ansiolíticos, conseguir tranquilizarse *un poco* y poder *dormir mejor*.

La mayoría refiere no sentir una gran diferencia en su forma de ser, aunque algunos señalan que al tomar psicofármacos vuelven a ser como eran:

“*Me cambia, pero igualándome con lo que yo era, me siento bastante más parecida a la época de no sufrir*”. (Lola, M55)

Otros sí perciben que dejan de ser ellos mismos:

“*No sé porque me da la sensación de que no te deja ser tú, te convierte en algo que no eres, aunque sea en el tiempo de la medicación. Algo que te convierte en algo que no eres…*”. (Eduardo, H26)

Aunque se trata más de un temor que de un cambio concreto:

“*Te quedas un poco pensando, hoy me siento así, pero me siento así porque me siento así, o por la medicación o por qué. Llega un momento que tengo la sensación de perder mis baremos, de perder el foco de referencia de quién soy yo y dónde estaba*”. (Diana, M28)

El control (o su ausencia) aparece con frecuencia en las narrativas de los pacientes:

“*Desde que estoy con pastillas controlo un poco los brotes, pero es como si se quedase dentro*”. (Concepción, M55)

Aunque los fármacos se ven como un medio para controlar las emociones, existe también la sensación de no ser capaz de hacerlo por uno mismo. Marta (M21) explica durante la consulta que necesita un trabajo, está de prácticas y se agobia porque quiere dejar de depender de sus padres, y quiere controlar la ansiedad por sí misma, sin tomar pastillas. Carlos (H47) llega a su primera consulta de psicología tras un tiempo en tratamiento con psiquiatría porque se ha enterado de que existen terapias psicológicas y quiere controlar sus emociones y sentimientos, quitarse la ansiedad y dejar la medicación. Le gustaría ser un poco libre: “*ser yo y controlar*”.

Diana (M28) empezó con una depresión reactiva al estrés y la alta competitividad de su trabajo como teleoperadora erótica. En el grupo de apoyo mutuo explica todo lo que toma: “*Zymbalta, Orfidal, Deprax, Enoltril y hace poco me han dado otra, abilin o algo así, es un antipsicótico que me ha dado peor rollo todavía*”. Otra participante del grupo, Beatriz, le pregunta si con tantas pastillas se siente como antes:

-“*No creo que actúes ni estés igual de espabilada*”. (Beatriz, M35)

-“*A veces me siento muy rara, como muy ajena*”*.* (Diana, M28)

Cuando habla, su discurso no se da en primera persona. Cuando le preguntamos algo durante las sesiones grupales -nunca habla si alguien no se dirige directamente a ella- casi siempre responde que su pareja o algún familiar han insinuado algo similar, pero siempre es algo que le sugieren desde fuera. La combinación de cinco psicofármacos no ha hecho que se encuentre mejor que hace un año, cuando la entrevisté, sino peor. La respuesta de la psiquiatra en cada sesión es aumentar la dosis o añadir un nuevo fármaco. Cuando meses después interrumpe el tratamiento y vuelve el grupo, su discurso ha cambiado: es mucho más lúcido y ella está más consciente de lo que la sucede.

Si junto al control aparece la idea de la desconexión y que “no afecten tanto las cosas”, esa desafección también es percibida por algunos participantes como extraña y como ajena, en el sentido de no ser uno mismo:

“*La primera vez me dieron Esertia al principio no notaba nada y luego poco a poco que las cosas me afectaban menos, pero las buenas y las malas*”. (Diana, M28)

[Al dejar la medicación] “*me siento, jolín, que vuelvo a ser yo. Porque te limitan mucho las pastillas, pierdes sensibilidad en las situaciones, eres un poco robot. Si una película me daba ganas de llorar a moco tendido, con las pastillas nada, o al revés igual no puedo echar una carcajada, me dejan como más frenada. Lo siento como si pasara... como si ni fu ni fa*”. (Concepción, M55)

La pérdida o la disminución de la libido y la imposibilidad de llegar al orgasmo o de eyacular son un lugar común en la consulta entre los consumidores de psicofármacos. La alternativa con la que se suelen encontrar es añadir alguna otra medicación, en ocasiones con interacciones o nuevos efectos secundarios, por lo que suelen terminar por asumir la ausencia de sexualidad, lo que en el grupo se comenta como un aspecto más del deterioro de la relación de pareja.

Otros efectos secundarios pueden ser una “pancreatitis” o que “*engordas, te ves mal físicamente*”, “*mareos que parece que me voy a desmayar*”, o “*que las piernas no me sostienen*”. También se señala que a veces se caigan las cosas, cólicos en el hígado o los riñones, tener pesadillas, que “*te confundan con un yonki*” por cómo te mueves por la medicación o una de las quejas más frecuentes, estar “*como muy aplatanado*”, “*como un zombi*”. Esta figura se repite en varios entrevistados: “*Vas como un globo, como un zombi*”; “*como un zombi, como dopada*”. La expresión condensa los dos elementos que acabamos de ver: el miedo a la ausencia de control y el no estar del todo vivo o que no te afecten las cosas.

Los efectos difieren de una persona a otra. La psiquiatra en la consulta lo explica así a sus pacientes: “*En psiquiatría vamos un poco a ciegas. Hay que ir probando porque lo que le va bien a uno no le va bien a otro*”. En este sentido, en el grupo, los participantes esperan tener los mismos efectos ante los fármacos para después descubrir que estos varían de una persona a otra. También aparecen dudas sobre qué es efecto de la medicación o del trastorno. Las quejas son continuas, aunque a menudo el profesional no le da importancia, simplemente se recodifican en el informe como “*rumiaciones en torno al tratamiento*”, convirtiéndolas en un síntoma más de la enfermedad:

“*La última vez fue un poco rarillo: es que parece que no te quieres tomar las pastillas. No me las quiero tomar, pero me las voy a tomar si es necesario, pero no sé, le decía más que nada los efectos secundarios y que no me estaban sentando bien. Y me cambió un poco: ‘bueno, te la cambio, pero no estoy convencida de que te esté sentando mal, sino que tú crees que te está sentando mal y por eso te sienta mal’*”. (Diana, M28)

Los propios participantes tienen esa duda:

-“*Yo lo que pasa es que no tengo mucha memoria, he perdido un montón, se me olvida, y de concentración fatal y tengo unos problemas porque luego no me acuerdo ni de lo que he dicho. Por el tratamiento o por a la depresión, no sé*”. (Beatriz, M35)

-“*Sí he notado durante mis épocas cuando tomaba pastillamen, lo de estudiar, lo de retener, tela, a mí por lo menos me costaba. Me decían que eran imaginaciones*, [risas], *sí, sí, qué va, qué va*”. (Hans, H45)

-“*¿Y eso puede ser por la depresión, que se te olvide todo y la concentración o eso es porque cada uno tiene la cabeza así ya como despistada?*”. (Beatriz, M35)

-“*Yo lo he notado bastante, me generaba ansiedad el olvidarme*”. (Hans, H45)

-“*A mí me está empezando a generar*”. (Beatriz, M35)

-“*Me generaba como una insatisfacción y frustración dedicarle horas y horas al estudio y que no me resultara. Incluso me deprimía... Te quedabas envuelto en una especie de burbuja, y en la primera etapa esa especie de adormidera era interesante porque se te olvidaban los problemas, pero llega un momento en que tú quieres volver al ruedo, no quieres quedarte en la burbuja*”. (Hans, H35)

### La consulta como negociación

En la consulta en psiquiatría se repasan los síntomas -en su mayoría signos fisiológicos- que dan lugar a un ajuste de la medicación. Se valora el nivel de activación, el sueño, el ánimo, el apetito y el nerviosismo junto a los posibles efectos secundarios de los fármacos como problemas de visión o de libido, mareos, dolor de cabeza u otros efectos controlados mediante analíticas con datos sobre el funcionamiento del hígado o el nivel de colesterol. En función de múltiples variables se puede aumentar o disminuir la dosis, cambiar el momento de la toma, añadir pastillas para contrarrestar los efectos secundarios o cambiar de fármaco, siempre con la posible pérdida de beneficios del actual. Esta dinámica implica continuas decisiones y renuncias sobre la medicación a modo de balanza costes-beneficios.

Es común probar medicaciones hasta que se da con la que sienta bien. Carlos (H45) pasó por cuatro: la primera le producía mareos, la segunda “*estar demasiado colocado*”, la tercera le daba fuertes dolores de estómago y la última le produjo paralización facial. Algunas personas llegan a desistir, otros en cambio aguantan.

Los consumidores de psicofármacos tienen un papel activo en todo el proceso de decisiones respecto a los fármacos. Suelen ser ellos los que plantean querer dejarlos o reducir la dosis, con o sin supervisión, aunque no todos los profesionales son igual de flexibles. En algunos casos, supone toda una negociación:

“*Digo, hasta aquí he llegado, yo creo que estoy fuerte. No se fían del todo, no me las quitan ahí anda, pero ya me empiezan a bajar* […] *Antes de ir al médico, me había quitado media ya, ya llegué sin. Y me dijo: bueno si ya te la has quitado, pues nada*”. (Concepción, M55)

También es común que no solo la decisión sino la propia retirada comience antes de la consulta y la supervisión médica. Varios pacientes refieren conductas como la de Diana:

“*Llevaba un año y medio y me lo iban subiendo, y subiendo, y subiendo, empezamos por 5 mg, íbamos ya por 20, llevaba un año y medio y era… no veo que avance, y si no avanzo no me hace gracia estar tomando estas pastillas. Sí, me da mucho miedo engancharme. Te lees los prospectos y dices: madre mía, qué me están dando. Me cambiaba de casa, estaba más animada, y llevaba dos días que se me había olvidado, y lo dejé*”. (Diana, M28)

La medicación se convierte en sinónimo de malestar, de su cualidad y su cantidad, por lo que es común escuchar en la consulta, como respuesta a la pregunta sobre cómo se encuentra una paciente: *“Llevo un mes a base de orfidales, hoy ya llevo tres”.* La toma de medicación se relaciona con estar enferma y la interrupción con estar bien. Clara (M26) habla de ella como enferma durante la época en la que estaba medicada, pero ahora, que consigue controlarse sin tomar medicación, no se considera así.

Autonomía, dependencia y cronificación

“*Un trastorno crónico es un negociazo*”. (Clara, M26)

La principal preocupación de las personas participantes con relación a los fármacos es la dependencia que generan. Hans lo explica así:

“*La autoestima crece si puedes controlarlo sin tener que tomar nada, porque dices, soy capaz. La idea es, vale, tengo estas carencias, que todos tenemos unas ciertas carencias en un sentido o en otro, joder, trabajando soy capaz de solucionar el problema. No tengo que acudir a química, que tiene además sus efectos secundarios, para no sé qué tipo de procesos*”. (Hans, H45)

El valor que se le da al consumo de drogas legales e ilegales también supone una imagen negativa de la dependencia de sustancias. Muchos refieren que se sienten “*como un yonki*”. Patricia explica por qué no le gusta la medicación: “*Me parece una droga, pero droga, droga, droga, de dependencia de verdad*”; o Carlos (H47): “*Ay, que me falta la pastillita... Tienes dos problemas: la ansiedad y la adicción*”. Varios participantes refieren haber sentido síntomas físicos al retirar la medicación, de manera que el síndrome de abstinencia también se ejemplifica como similar al de otras drogas:

“*Sentí respuesta física por dejarlo. Empecé con una temblera, fui al médico, iba como drogada, con las pupilas dilatadas*”. (Clara, M26)

Casi todos los participantes viven con miedo y preocupación el momento de dejar la medicación, y algunos tienen ya asumido que nunca la dejarán. Cuando una persona se encuentra mejor no sabe si tras dejar la medicación volverá a encontrarse en el punto de partida que le llevó a empezar el tratamiento, por lo que no existe la certeza de una mejora real del malestar:

“*Y sí, hacen las pastillas, pero a ver, la prueba de verdad es cuando no las tome. Es un engañabobos, la pastilla no cura, no soy diabética. La pastilla me va frenando, me va dejando sobrevivir medianamente con un poco de dignidad, pero si cuando las dejas vuelves otra vez al bucle, ¿de qué me han servido? No me han servido de nada*”. (Concepción, M55)

Muchos participantes mejoran al principio del tratamiento y vuelven a encontrarse mal unos meses después de dejar la medicación. Las recaídas no solo son frecuentes, sino que “*son peores*” por la “*pérdida de confianza*” que suponen. En la misma línea del ser capaz y de hacerlo por uno mismo, Beatriz asume el no poder dejar las pastillas como un signo de su debilidad:

“*Yo lo tengo claro porque lo he intentado dos veces y no* [...] *yo quiero dejarlo por los efectos secundarios que tienen todas las pastillas, y porque mi madre está tó pesada que tengo que ser fuerte yo misma, que sacarlo yo misma. Porque ella también ha tenido algunos indicios de depresión cuando se separó y ella pudo ella sola. Pero no todos somos igual, mamá, si todos fuéramos igual no existirían las pastillas. O hay gente que es más débil, y otros son más fuertes. He intentado dejarlo, por eso, pero a los dos o tres meses he recaído otra vez*”. (Beatriz, M35)

Apenas existe información por parte de los profesionales sobre las expectativas del tratamiento y el posible curso del padecimiento. Se recomienda mantener el tratamiento un tiempo pese a la mejoría, sin dejar claro si habrá una remisión. Los psiquiatras suelen restar importancia a la toma de medicación, como explica Lola, con diagnóstico de trastorno reactivo a una difícil situación en el lugar de trabajo:

“*Me preocupa, no me gusta, pero luego como hablo con ellas* [la psicóloga y la psiquiatra] *y les digo que no me gusta tomar medicinas y me dicen que no es nada y que me está aportando calidad de vida en el trabajo, y entonces pues supongo que el cuerpo también se deteriora sin tomar*”. (Lola, M55)

La cotidianidad de las personas del centro de salud mental termina marcada por los sucesivos intentos para bajar o eliminar la medicación:

“*Aquí sigo con la lucha de a ver si me pueden bajar la dosis y quitarme, pero en cuanto dejo de tomar algo me pongo de los nervios*”. (Blanca, M49)

### ¿Qué me pasa? La atribución del malestar

En los relatos de las personas entrevistadas apenas aparece la cuestión cerebral. Ni durante la observación, ni de forma espontánea en las entrevistas se expresa como una explicación al padecer. En la observación, tan solo en una ocasión, la esposa de un paciente le preguntó a la psicóloga que cómo sabían que le faltaba una vitamina en el cerebro, si ni siquiera le habían hecho pruebas. El marido indicó que se trataba de la serotonina. Esta es la única variable que aparece en el grupo, cuando se discute acerca de la posibilidad de que la depresión tenga una causa biológica:

“*El tema de la serotonina, están las sinapsis, eso dicen, parece que está estudiado, la recaptación, el que sueltes más serotonina te lo fomentan las pastillas. Las personas que se deprimen, supongo que no serán todas, si no, biológicamente recaptan mucha serotonina, eso parece ser…*”. (Hans, H45)

A pesar de que la mayoría toman fármacos y notan efectos de mejora, eso no supone una atribución automática al malestar como algo biológico. Tan solo Hans, que conoce algo mejor el funcionamiento de los antidepresivos sugiere esa relación.

“*Si no es biológico, ¿para qué estamos tomando lo de la serotonina? Lo lógico sería pensar que nosotros recaptamos mucha serotonina, se queda allí en la sinapsis y a otros le fluye que no veas y están tan contentos, parece que con los fármacos es así*”. (Hans, H45)

No obstante, pese a reconocer la importancia de los problemas sociales en la aparición del malestar, al hablar de sus experiencias particulares, los participantes acaban remitiéndose a algo que les es constitutivo, cierta tara o falla personal. Pese a que los factores sociales o laborales aparecen de forma recurrente en las narrativas, lo hacen como factores secundarios que agravan su condición crónica de vulnerabilidad. Aunque al hablar de conocidos o amigos, la atribución es distinta, cuando se trata de elaborar un relato propio, la explicación siempre se acompaña de las limitaciones personales, que aparecen como determinantes. Pedro, sindicalista con graves problemas laborales, dijo de forma firme la primera vez que hablé con él: “*Lo que está claro es que lo mío fue un proceso reactivo*”. No obstante, cuando en el grupo se discute acerca de la cronicidad del malestar expone:

“*Quizás yo ya tenía algún puntito, mi madre era depresiva, yo en estos 6 años he mejorado, pero tengo temporadas que vuelvo a recaer… Hay personas que están tocadas, o estamos tocados, que somos más propensos a que esto no desaparezca por completo, pero creo que, en mi caso, tengo algún defecto de fábrica*”. (Pedro, H58)

Del mismo modo, Lucas, con un proceso de estrés laboral, apunta a esa responsabilidad por lo que les pasa:

“*Creo que estoy predispuesto para tener ansiedad, por eso es como vivo la vida. Porque ya lo llevo dentro, me he forjado esa personalidad*”. (Lucas, H47)

Las sucesivas recaídas se ven como sinónimo de falla individual y de ese modo refuerzan el rol de enfermo. Pedro tiene 58 años, se afilió a Comisiones Obreras (Confederación Sindical de España) con 18 años y dedicó toda su vida a la lucha sindical. Tras perder el juicio con su empresa seis años atrás -en el que el sindicato ni siquiera se presentó- tuvo un intento de suicidio y le dieron la incapacidad laboral, ahora permanente, por depresión mayor. Su imagen como sindicalista dio paso a la de un enfermo incapaz de trabajar.

Al analizar en profundidad a las personas entrevistadas que señalaron el trabajo como foco principal de malestar, la mayoría presentaba conductas de resistencia, activas, frente a situaciones que consideraban injustas. De los once entrevistados que plantearon esa queja tan solo dos no mencionaron conductas explícitas de resistencia. Es decir, el padecimiento no venía derivado de la aceptación de las condiciones laborales, sino de su rechazo, y de los conflictos que ese rechazo a las condiciones laborales ocasionaba con los superiores. Varios participantes tan solo tomaban medicación mientras duraba su jornada laboral, y dejaban de tomar cuando descansaban los fines de semana y en periodo de vacaciones. La principal razón por la que tomaban medicación era para soportar la presión, la mala actitud o las represalias. El siguiente registro de la psiquiatra, resume la cotidianidad de las consultas de los pacientes con problemas laborales:

“*Acude a la consulta con sintomatología ansiosodepresiva a raíz de conflictos a nivel laboral en 2010, sintiéndose desbordada, con ansiedad con múltiples síntomas somáticos,* [...] *así como rumiaciones sobre el problema laboral. Se reinstauró tratamiento antidepresivo con mejoría progresiva de la sintomatología y evolución favorable, siendo también más capaz de distanciarse de los problemas que se mantienen en el ámbito laboral*”. (Diario de campo)

En la consulta, las palabras de ánimo de la psiquiatra revelan la relación entre intervención y conformidad: “*La situación está igual, pero tú estás mejor*”. Además de soportar situaciones injustas, en muchos participantes se interioriza la culpa:

“*Tú no te presentas y dices hola, soy Clara y tengo problemas de estrés, si me presionas en exceso. Pero si ya tú eres una persona que sufres y te tratan así...*”. (Clara, M26)

## DISCUSIÓN

La subjetivación analizada en nuestro estudio tiene lugar más en forma de problematización que de interiorización automática. En la línea de los análisis del último Foucault, que se interroga por la forma no determinista en que las personas se relacionan con lo que denomina las tecnologías del yo^43^^,^^44^, la aproximación al encuentro médico sucede como una negociación, con una recepción ambigua. Esta ambivalencia pone en entredicho la visión de la medicalización como un proceso acogido con entusiasmo por las personas, y revela el malestar que el propio consumo de fármacos conlleva.

Podemos entender que las dos lógicas presentes en la intervención (cerebral y terapéutica) operan de manera simultánea porque el tipo de *self* que ambas proponen se corresponde con la subjetividad neoliberal, es decir, con la forma en que se nos moviliza en nuestra sociedad. Se trata del ideal de una persona autónoma, con iniciativa, que se acepta incondicionalmente sin necesidad de aprobación y que controla las emociones negativas, que maneja riesgos y evalúa su ánimo, su humor y sus reacciones fisiológicas^45^. La lógica neoliberal implica favorecer la competitividad en las empresas, pero también incitar al individuo a convertirse en una empresa^46^. En la subjetividad revelada por la narrativa de los participantes, se vuelven centrales aspectos como el miedo a la recaída y a los efectos secundarios, un ideal de autonomía difícil de lograr cuando uno siente que el cambio no es consecuencia del trabajo personal y el sentimiento de responsabilidad por lo que a uno le sucede.

### El pharmakon y la consulta como negociación: autoatención y responsabilidad individual

La principal característica destacada por los participantes es la relación ambivalente que establecen con los fármacos que consumen. Martin^47^ se refiere a esta cualidad con el antiguo término griego *pharmakon*, que significa simultáneamente remedio y veneno. Los fármacos, a la vez que mitigan parcialmente el malestar de las personas, generan nuevos problemas como los efectos secundarios y la dependencia, produciendo un nuevo malestar^21^. Frente a la idea de la posibilidad de mejorar o de diseñar un *self* a voluntad del consumidor, los participantes del estudio no tratan de ser felices u olvidar sus problemas, ni de convertirse en personas diferentes. Hablan de supervivencia y de volver a entrar en el circuito productivo, aunque sea a niveles mínimos y a costa de numerosas renuncias. Entre ellas, la capacidad de que te afecten las cosas, la concentración para estudiar o el deseo sexual. Cuando estas problemáticas se exponen en la consulta a menudo se minimizan, e incluso resignifican como “*rumiaciones en torno al tratamiento*”, convirtiéndolas en un síntoma más de la enfermedad. Pero la realidad es que no es posible aislar los efectos secundarios de la experiencia de la medicación^31^.

Los consumidores de psicofármacos hacen continuos balances y toman decisiones sobre la medicación. Aunque desde el enfoque biomédico se suele criticar la automedicación, esta es coherente con el tiempo de espera entre citas, con la frecuente prescripción a demanda (ansiolíticos) y con el escrutinio de síntomas y reacciones que tiene lugar en la consulta. Mientras que el personal sanitario se suele referir a la automedicación como algo negativo, desde el sector se impulsan de forma continua actuaciones que van en esa dirección^48^.

Según Rose^5^ la lógica del desarrollo personal y la autenticidad de la cultura terapéutica también existen en la intervención farmacológica, ya que los fármacos son prescritos y consumidos como una forma de tomar control sobre uno mismo. Es decir, no entrarían en contradicción con las tecnologías del yo descritas por Foucault^49^ como las formas en que los individuos se experiencian, entienden, juzgan y se conducen. Los fármacos también serían tecnologías para la conducción de la relación con uno mismo en forma de autonomización a través del rol activo de los consumidores. El ideal de control aparece con frecuencia en las narrativas de los pacientes, aunque siempre como algo a alcanzar, ligado a la autonomía. A pesar de que los fármacos se ven como un medio para aumentar el control sobre las reacciones emocionales, coexisten con la sensación de no ser capaz de hacerlo por uno mismo, con la percepción de incapacidad de manejar las emociones de forma autónoma^50^, y con uno de los efectos más comunes descritos por los consumidores en este y otros estudios, el ir como un zombi, como sinónimo de no tener conciencia^51^.

La primera característica de la subjetividad farmacéutica encontrada en los participantes sería entonces el establecimiento del autogobierno a través de la autoatención. El manejo del *pharmakon* -la doble naturaleza de los fármacos como beneficiosos a la vez que nocivos- implica una constante monitorización y toma de decisiones en forma de balanza de costes-beneficios.

### Cronificación y vulnerabilidad

La principal preocupación de las personas entrevistadas es la dependencia que generan los fármacos. Existe una contradicción entre el ideal de autonomía que caracteriza la subjetividad neoliberal, la dependencia farmacológica, y la sensación de no conseguir la mejora por uno mismo. Los sucesivos intentos de los participantes para bajar o eliminar la medicación sin éxito suponen que, en lugar de la cura, se produzca una cronificación del malestar en un alto número de personas. El caso que es más visible es el de las pastillas para dormir, que de forma paradójica terminan produciendo insomnio^52^, pero también los antidepresivos tienen un efecto similar^53^^,^^54^. Esto supone un modelo de enfermedad por el que el individuo que se recupera está en riesgo de futuras recaídas, y la medicación se receta tanto para el riesgo como para la condición. Los consumidores de psicofármacos entrevistados se ven como enfermos, en riesgo y dependientes. Las sucesivas recaídas pasan a entenderse como sinónimo de falla individual.

El hecho de que la intervención gire en torno a la medicación ha producido, en parte, que el malestar sea percibido, interpretado y vivido como una dolencia médica, a menudo de forma independiente de la intensidad o el origen. Charmaz^55^ describe, como una de las principales causas de sufrimiento en las personas con enfermedades crónicas, la pérdida del *self*. Las antiguas imágenes desaparecen y el padecimiento se vuelve el centro de la vida. La imagen de muchos de los participantes en nuestro estudio es la de enfermos, incapaces de trabajar, tolerar el estrés, etc. Un nuevo *self* marcado por la vulnerabilidad, la falla y el riesgo. Martínez-Granados *et al*.^56^ también encontraron en pacientes con diagnósticos cronificados en salud mental que la medicación aparecía como símbolo de vulnerabilidad y fragilidad. Se trata, además, de una vulnerabilidad que se acentúa en los procesos de retirada y/o disminución de medicación.

Los ideales de autonomía, trabajo y responsabilidad individual aparecen como el reverso al *self* vulnerable del rol de enfermo. En una sociedad que prioriza el hacer frente al ser, no poder realizar las tareas convencionales dificulta mantener una vida con sentido^55^. Concebir nuestra biografía en términos de enfermedad produce una reflexividad permanente en la que nuestros *selves* son continuamente problematizados y patologizados. La vida se convierte en continuas revisiones y mejoras.

La segunda característica de la subjetividad farmacéutica encontrada en nuestro estudio es la cronificación del tratamiento relacionada, por un lado, con la dependencia que generan los fármacos y, por otro, con la noción de riesgo en torno a la que se articula el tratamiento. La sensación de estar en permanente riesgo -de recaer o de empeorar- es otro rasgo de la subjetividad farmacéutica.

### Reificación del malestar

En el contexto del estudio, la intervención en la consulta de psiquiatría se limita a la prescripción de psicofármacos. La relación que establecen las personas estudiadas con los fármacos es compleja y ambivalente pero, en general, subrayan que se abusa de la prescripción frente a la terapia. El tratamiento es el que guía toda la intervención, desde el diagnóstico hasta el propio encuentro clínico. La centralidad de la prescripción conforma el imaginario referente a las atribuciones del malestar y a las posibilidades que se abren cuando se trata de abordarlo. Aunque la explicación del padecer trascienda lo biológico, la práctica termina por marcar su abordaje, que en la consulta aparece como una cuestión de autoregulación fisiológica entre síntomas y efectos secundarios. Como describe Martínez^57^, si modifico mi cerebro no es necesario modificar el mundo, de forma que acabo primando el *self* sobre el mundo social. 

A diferencia de los estudios en los que los consumidores de psicofármacos sitúan en el cerebro el origen de su malestar^25^^,^^58^, en nuestro contexto apenas aparecen narrativas cerebrales, ni siquiera coexistiendo con otras, como se han encontrado en otros análisis en nuestro país^59^. El sistema público de salud limita la mercantilización y el marketing en España, que se dirigen a los profesionales en lugar de a los pacientes, al contrario de lo que ocurre con los anuncios directos al consumidor en EEUU^37^. Factores culturales, sociales y contextuales determinan entonces el imaginario que se construye en torno al uso de fármacos y en torno a los diagnósticos y al padecer mental.

No obstante, cuando los participantes hablan de sus experiencias particulares, siempre acaban remitiéndose a algo que les es constitutivo, a cierta tara o falla personal. La explicación de corte más social queda cortocircuitada por la lógica biomédica, mediante la generación del rol de enfermo. El padecer no se codifica en términos exclusivamente biológicos, pero sí queda articulado en torno a la figura de la vulnerabilidad, de la debilidad, del déficit. La angustia personal se plantea como un problema que debe tratarse mediante soluciones farmacéuticas alineándose con los imperativos neoliberales de autooptimización, en lugar de abordar las causas estructurales o sociales de la angustia. 

Además, el malestar muchas veces conforma formas aisladas de resistencia a las condiciones de vida, por ejemplo, en el trabajo. Y esas resistencias o cuestionamientos traducidos al lenguaje biomédico son codificadas como fallas individuales, descontextualizadas y vaciadas de su contenido simbólico, singular y social. La intervención aparece, en estos casos, como una forma de reificación en cuanto que interviene sobre subjetividades que se han salido de lo normativo.

La tercera característica encontrada sería entonces la reificación, que supone la instauración de un modelo explicativo del malestar que limita los factores sociales a meros agravantes, para poner en el centro de la justificación del padecer una predisposición individual, una forma de déficit o tara por la que la persona está menos capacitada para hacer frente a las dificultades.

### Limitaciones y contexto actual

Es importante remarcar que, al señalar los efectos negativos que pueden tener los psicofármacos, no se pretende demonizar a los individuos que los toman o los beneficios que pueden tener^60^. Además, a pesar del valor que tienen las narrativas de los consumidores de psicofármacos -en el contexto concreto de un centro público de salud mental, en Madrid, en la anterior crisis económica- la principal limitación del estudio es, precisamente, su acotación contextual. Por un lado, la crisis que vivimos ahora es distinta, con unas tasas de desempleo menores, y con el encadenamiento de años en los que la mejora en las cifras macroeconómicas no va de la mano de las mejoras en las condiciones de vida. Esto hace pensar que es posible que la sensación de responsabilidad individual por el fracaso pueda ser menor en el contexto actual. Por otro, pese a que estudios recientes en EEUU siguen encontrando un aumento en la tendencia a articular el sufrimiento en términos neurobiológicos en lugar de a acontecimientos externos de la vida^61^, habría que evaluar cómo la pandemia ha podido impactar en las narrativas en torno al padecer, al poner en primer plano los efectos del contexto en la salud mental. Sería necesario, entonces, seguir interrogando y escuchando las voces de las personas que consumen psicofármacos a partir del nuevo contexto que habitamos. 

## CONCLUSIÓN

La intervención psiquiátrica en el contexto del estudio implica cierta unidireccionalidad en la prescripción y en la relación con el profesional. No solo no existe una explicación neurobiológica en los participantes, tampoco existe una demanda clara de psicofármacos que se ven, en general, como un mal menor para “*sobrevivir*” en ausencia de la posibilidad de realizar una terapia. La intervención produce una forma de subjetividad, que podemos denominar “farmacéutica”, caracterizada por su ambivalencia y por la necesidad de negociar ajustes continuos para minimizar los efectos secundarios y para evitar caer en la cronificación. 

Como elementos clave de esta subjetividad, encontramos la reificación, por la que prima la sensación de estar en déficit; el autogobierno, que implica una constante monitorización que acentúa el rol de enfermo; y la cronificación, que supone percibirse como siempre en riesgo y, por tanto, vulnerable. Frente a la noción de medicalización, que presenta un proceso unidireccional y sin matices, se ha preferido emplear el término “subjetividad farmacéutica” para dar cuenta de la complejidad de los efectos de la intervención. En la subjetividad resultante se vuelven centrales aspectos como el miedo a la recaída y a los efectos secundarios; la autonomía, difícil de lograr cuando uno siente que el cambio no es consecuencia de los propios logros; y la responsabilización por el sufrimiento y el autogobierno, elementos centrales de la subjetividad neoliberal. 
