# Supplementary material for: Subjetividad farmacéutica en tiempos de crisis en Madrid: Entre la supervivencia, la cronificación y el “Debo ser yo”
Source: Salud Colect. 2025 Mar 6;21:e5563. [Article in Spanish] doi: 10.18294/sc.2025.5563 (PMC12303866; doi:10.18294/sc.2025.5563)
Supplement: Translation

# Translation: Pharmaceutical subjectivity in times of crisis in Madrid: Between survival, chronicity, and “It Must Be Me”

**Authors:** María Reneses

**Affiliations:** 1 PhD in Anthropology. Postdoctoral Researcher, Instituto de Investigación Tecnológica, Escuela Técnica Superior de Ingeniería (ICAI), Facultad de Ciencias Humanas y Sociales, Universidad Pontificia Comillas, Madrid, Spain. mariareneses@gmail.com

**Keywords:** Mental Health, Psychotropic Drugs, Metabolic Side Effects of Drugs and Substances, Spain

## Abstract

In recent years, mental health has gained significant relevance, accompanied by a gradual reduction in stigma. As a result, more people are understanding and analyzing their suffering in psychological terms. However, for many individuals accessing public mental health services in a city like Madrid, medication remains the primary treatment option. This study aims to analyze the type of subjectivity produced through this form of intervention, based on the findings of an ethnographic research conducted between 2012 and 2014. The research included observation in mental health center consultations, 19 in-depth interviews with psychotropic drug users, and a reflection group with periodic meetings. Key findings highlight ambivalence towards medication and the need for continuous adjustments to minimize side effects. The resulting subjectivity revolves around central aspects such as the fear of relapse and side effects; autonomy, which conflicts with the notion of not trying on one’s own; accountability, feelings of vulnerability and self-governance, core aspects of neoliberal subjectivity.

## INTRODUCTION

The way we perceive mental health, psychological distress, and its treatment depends on our sociohistorical context and, therefore, is constantly evolving.^1^ Not only have intervention methods changed over time, but society’s own perceptions have also shifted from the era of confinement to psychiatric reforms. According to medical anthropology and the anthropology of social suffering, our relationship with distress, our responses to it, and our valuation of mental health and mental health care are shaped by new forms of governance. When categories and practices from everyday common sense are replaced by biomedical rationality, the core values and dominant forms of contemporary subjectivity are transformed.^2^

First, although stigma persists, particularly toward vulnerable groups, there has been a significant destigmatization of mental health issues.^3^ While this trend predates the pandemic, it appears to have intensified in its aftermath.^4^ Alongside destigmatization, neoliberal society increasingly promotes the management, modulation, and enhancement of human capacities, as well as the optimization of the mind and emotions.^5^ In addition to self-help books, coaches, and psychologists, the consumption of prescription drugs - prescribed by primary care physicians and psychiatrists - has proliferated as another tool for improving emotional well-being.

As a result, individuals are increasingly inclined to define psychological distress or discomfort as a mental health issue and to seek professional help for it. Within this context, it is crucial to analyze how the main form of intervention in Spain - at least within the public healthcare system - psychiatric medication, affects and shapes individual subjectivity. To this end, this article presents a portion of the empirical material collected for the author’s doctoral dissertation.^6^

While the destigmatization of mental health and the growing demand for additional resources may be considered positive developments, it is essential to examine how this centrality of mental health affects individuals who have limited access to therapeutic resources. This study analyzes the characteristics of pharmaceutical consumption and the resulting pharmaceutical subjectivity, foregrounding the voices and experiences of a group of consumers who have not been as extensively studied as those diagnosed with severe illnesses-namely, patients with milder diagnoses, whose suffering is largely reactive to the crisis and conditions of precarity.

The study was conducted within the context of the previous economic crisis (2012-2014), during which unemployment and evictions were widespread across Spain. The inadequacy of social safety mechanisms meant that a significant portion of the population fell below the poverty line,^7^ particularly in Puente de Vallecas, one of the areas most affected by the crisis in the Madrid region.^8^ At the time (as remains the case today), Puente de Vallecas was one of Madrid’s districts with the lowest per capita income, the highest increases in unemployment and housing costs, and the worst indicators of perceived health, life expectancy, and income levels. Moreover, the district is highly dependent on public assistance, with minimal private or mixed healthcare coverage.^9^ The mental health center where the ethnographic research was conducted was not only overwhelmed by increasing demand and budget cuts but also faced a looming threat of privatization. Although privatization ultimately did not take place, professionals’ contracts, which were initially six months long at the beginning of the study, were gradually reduced, first to three months, and later to just one month.

### Psychotropic drug consumption and social determination

Although the consumption of psychotropic drugs in Spain has seen a slight decline in recent years,^10^^)^ likely due to relative economic improvement, according to the United Nations, in 2022,^11^ Spain ranked among the countries with the highest psychotropic drug consumption in the world, leading in benzodiazepine use. Furthermore, a 2023 study found that most psychotropic drug users among Spain’s salaried population attributed their consumption to work-related reasons (11% stated this, compared to 3.6% who did not).^12^ In recent years, studies in Spain have consistently demonstrated the effects of socioeconomic conditions on mental health. The use of anxiolytics and antidepressants is positively correlated with belonging to a lower social class, being unemployed, experiencing housing insecurity, or being responsible for the care of a disabled person.^13^^,^^14^^,^^15^ Regarding gender, women not only continue to report poorer mental health but also experience overdiagnosis and medicalization, which includes a higher prescription rate of psychotropic drugs.^16^

However, psychotropic drug consumption data are complex and cannot be attributed solely to epidemiological reasons. As Lakoff^17^ points out, this is not merely a case of contamination of pure science but rather a structured system of vested knowledge that deeply intertwines marketing with science within the biomedical economy. The increase in antidepressant consumption may indicate a rise in the prevalence of depression, or it may reflect a marketing strategy that promotes the use of antidepressants for anxiety instead of benzodiazepines^18^.

Moreover, the issue of overprescription, as well as the specific medications prescribed, is not solely driven by the pharmaceutical industry or by the biologicism that characterizes contemporary psychiatry. It is also a response to demand in the absence of alternative treatments. Prescription is a social act through which the physician acknowledges the patient’s suffering and demonstrates their willingness to help. Medications are perceived as the essence of medical practice, and, as a result, prescription is what patients expect from doctors.^19^ Given that global concern about mental health is on the rise, insufficient attention has been paid to sociocultural knowledge, both regarding mental illnesses and the effects of their treatment.^20^

### Pharmaceutical subjectivity

Pharmaceuticals exert effects not only through their chemical properties but also through the imaginaries constructed around them. They are both products of and contributors to human culture. As facilitators of self-care, they shape individuals’ thoughts and actions, influencing social life and contributing to what can be termed the production of pharmaceutical subjectivity.

This subjectivity is composed of both the self and the pharmaceutical imaginary, which function as two interconnected aspects. Jenkins^21^ defines *the self* as the sum of the processes through which an individual orients themselves in the world and in relation to others. The other element, the imaginary, refers to the cultural dimension that defines the conceivable possibilities of human life. The imaginary, which is not limited to consumers alone, encompasses feelings, fantasies, values, and ideals. In this sense, the marketing of a drug or a disease produces an imaginary that legitimizes certain behaviors or emotional states as appropriate while deeming others undesirable or subject to sanction.

According to Pazos,^22^ the formation of subjectivity involves processes of incorporation, understood as ways of acting and speaking, systems of dispositions or *habitus* that structure the experience of the self. The categories used to classify individuals ultimately produce new forms of personhood that did not previously exist.^23^

Nikolas Rose^24^ describes the phenomenon of progressive pharmacologization and its reflection in subjectivity as the neurochemical self. For Rose, this represents a shift from a psychological understanding of the individual to a somatic individuality, an individual increasingly conceptualized in biomedical terms, who seeks to modify, heal, or enhance themselves through interventions on the body. Emily Martin^25^ similarly argues that the pharmaceutical self internalizes not only pharmaceutical companies’ marketing strategies but also the ambivalences that consumers navigate due to side effects.

Finally, Biehl and Morant-Thomas^26^ draw on Deleuzian conceptions of subjectivity, understanding it not merely as a product but also as a producer of new social realities. This perspective moves beyond the traditional agency-versus-structure debate by considering both as affectively interconnected rather than mutually exclusive. According to these authors, subjectivity is more a process of becoming than a fixed structure. Biomedical interventions produce subjectivation by generating new ideas about what individuals are, what they should be, and what they can expect to become.

One of the most extensively researched areas regarding subjectivity produced in the medical encounter has been the relationship between medical interventions targeting women and the shaping of gender roles-for instance, through pharmaceuticals designed to alleviate menstrual pain^27^ or assisted reproductive technologies^28^. The effects of chronic treatments on subjectivity and body perception have also been studied, such as in the case of HIV antiretroviral therapy^29^ or diabetes medications.^30^

Research on the experience of psychotropic drug use among patients with severe diagnoses has identified several key characteristics. These include the impossibility of isolating unwanted side effects from the overall experience of medication, as well as the significance of contextual factors in shaping individuals’ perceptions of treatment.^31^ Studies have also highlighted institutional barriers that limit patients’ right to discontinue treatment-something that is often done autonomously using self-help strategies and social support networks, particularly to manage the so-called “withdrawal effect”.^32^

The following section will describe the experiences with psychiatric medication among a group of individuals characterized by having mild diagnoses, most of which are considered reactive to the economic crisis.

## METHODOLOGY

Between 2012 and 2014, I conducted fieldwork in three phases for my doctoral dissertation with individuals receiving care at the public mental health center in the Puente de Vallecas neighborhood. The first phase consisted of daily observation over two months in the consultations of a psychiatrist and a psychologist. In the second phase, I conducted 19 individual interviews with psychotropic drug users. The third and final phase involved a group composed of some of the interviewed individuals, which met periodically over the course of two years. Although the results primarily focus on the second and third phases, the study reflects the entire ethnographic process. The three-stage design allowed for distinct research tasks: the first phase of observation aimed to broaden the scope of inquiry and define the research object; the second phase of interviews focused on refining and formulating hypotheses; and the third, group-based phase was dedicated to testing those hypotheses.

Observation enables the comparison between what emerges in the clinical encounter and what is expressed outside of it. According to Kleinman,^33^ understanding subjective changes in specific historical contexts requires not only examining macro-political transformations but also analyzing the local worlds where cultural representations and collective processes manifest. Ethnography is a key methodology for such analysis, including within medical contexts.^21^^,^^34^^,^^35^^,^^36^^,^^37^ Ethnographic research allows for the study of self-formation patterns, the ways in which inner life and values are shifting, and how these transformations impact suffering and its interpretation.^26^ This approach also contributes to understanding the meaning of mental health and psychiatric care in this specific context.

The participants were selected based on findings from the observation phase. Most of the individuals seen in the consultations identified the onset of their distress with a recent event or situation, such as suffering related to waged labor, unemployment, or unpaid reproductive labor, particularly full-time caregiving for dependent individuals. For this reason, nearly all selected participants had one of these issues as their primary concern. However, one participant without a clear social precipitant was also included (Table 1), as he showed a particular interest in the effects of medication during consultations.

**Table 1:** Characteristics of the interviewed individuals. Madrid, 2012-2014.

No°	Fictional name	Gender	Age	Main demand/problem	Socio-laboral situation
1	Rocío	Female	49	Dysthymiax	Unemployed cleaner with fibromyalgia and a child with a disability
2	Concepción	Female	55	Care	Caregiver with financial problems (depends on her partner with whom she has issues)
3	Diana	Female	28	Work-related stress	Telemarketer
4	Matilde	Female	60	Care	Caregiver with financial problems
5	Beatriz	Female	35	Relationship and work problems	Salesperson
6	Eduardo	Male	26	Dismissal	Salesperson
7	Carmen	Female	40	Workplace harassment	Cleaner
8	Clara	Female	26	Work-related stress	Alternates between precarious contracts and unemployment in research
9	Pedro	Male	58	Dismissal after union struggle	Unemployed ex-unionist
10	Lucas	Male	47	Work-related stress	Cook
11	Blanca	Female	49	Work-related stress	Cleaner. Husband and daughter unemployed.
12	Hans	Male	45	Anxiety	No reactive situation
13	Marta	Female	21	Alternates between precarious work and unemployment	Can’t become independent, has to contribute money at home
14	Inmaculada	Female	31	Unemployment	Debts, cancer under treatment
15	Carlos	Male	47	Economic problems	Works, but doesn’t make ends meet
16	Isabel	Female	50	Work problems	Husband unemployed, ex-caregiver
17	Lola	Female	55	Work problems	Referred due to a workplace accident
18	Patricia	Female	38	Workplace harassment	No financial problems
19	Olga	Female	50	Unemployment	She and her husband are unemployed, depend on parents’ pension

Source: Own elaboration.

A total of 19 in-depth interviews were conducted. The selection of participants was based, on the one hand, on a certain representativeness, or what Guber^38^ refers to as the redundancy of social life. In this case, caregiving, labor-related issues, and unemployment emerged as dominant themes within the intervention practice due to their recurrence. On the other hand, ethnography, unlike other methodologies, is not limited to representation. As Han^39^ explains, singularity can also be of interest; therefore, in some cases, participants were selected without a clear triggering factor, following a case study approach rather than a probabilistic one.

All interviews were conducted outside the healthcare center to help participants separate contexts and avoid responses influenced by concerns about their treatment or the expectation of providing a more “scientific” answer. It was necessary to establish a distinct role from the clinical setting and emphasize that there were no right or wrong answers, the purpose of the interview was precisely to capture the elements that often remain outside the medical encounter. In the case of caregivers - most of whom were women - only two interviews could be conducted, both in the participants’ homes.

The interviews followed an open, ethnographic format, guided in part by the interviewee but with specific topics to be addressed. Given that the interviews dealt with intimate and often difficult-to-verbalize issues, the interviewer maintained an active and sensitive listening approach, paying attention to the variation between explicit and implicit elements in discourse.^40^

The mutual support and reflection group not only served as a source of empirical material but was also intended to benefit the participants and contribute a more propositional component to the research by exploring viable alternatives to biomedical interventions for distress. In this sense, it functioned as a form of clinical anthropology^35^ or as a locus of resistance.^41^ While the group was offered to all participants, four chose not to participate because they felt better, three due to scheduling conflicts, and one due to embarrassment. Beyond its empirical purpose, the group, which met over the course of two years, also had an ethical dimension, aiming to return some of the knowledge generated back to the participants. Additionally, it sought to contribute to the construction of more community-oriented alternatives, a demand that emerged in multiple interviews, as illustrated by Rocío’s comment:

*“Unless someone has a little self-respect and decides on their own, ‘I’m going to form a group with these people,’ but of course, those people need money”.* (Rocío, F49)

Observations were recorded in a field diary, and group sessions were recorded and transcribed only in selected excerpts. Interviews were fully transcribed and analyzed using categorical content analysis, that is, the text was broken down into units that were grouped into categories according to thematic areas.^42^ The categories identified were: drug effects, difficulty in identifying effects, disaffection, zombie effect, changes in self-perception, survival/improvement, side effects, challenges in discontinuing treatment, dependency, contradiction between dependency and autonomy, self-management, perception, stigma, and overprescription. Within this framework, direct quotes were included to illustrate key ideas. Finally, the data extracted through the categorical system served as the basis for conceptualization and interpretation.

The study was approved by the Ethics Committee of the General University Hospital Gregorio Marañón under approval code 319/11-IP, and all participants signed an informed consent form. To ensure confidentiality, all participant names have been anonymized.

## RESULTS

*“Now I’m high, I’m balanced. But it’s artificial, I want to get used to not feeling wrapped in cotton wool.”* (Hans, M45)

*“I’m losing confidence in myself... Taking the medication makes me feel like I’m no longer trying on my own.”* (Concepción, F55)

### Intervention as prescription

All patients who attended the consultation had already been prescribed anxiolytic or antidepressant treatment by primary care after a period of not experiencing improvement. From the very beginning, their relationship with medication was complex and ambivalent, but the prevailing perspective among them was that prescriptions were overused:

*“I think talking always helps more than pills; I’m not a fan of chemicals. Talking to someone and connecting is always better* [...] *Therapy is the best thing - if we could avoid taking pills through therapy.”* (Eduardo, M26)

*“It’s not just about taking a little pill and everything goes away. What we need is more therapy -less pills, more therapy. If we had received therapy before things got so bad, we wouldn’t have ended up in this state, needing medication. I’m sure some of us could stop taking the pills, or wouldn’t have needed them in the first place.”* (Concepción, F55)

Overprescription was described in terms of increasing dosages or the continual addition of new medications:

*“Every day they give you a new one, and you think: I’m going to end up taking 50 pills a day.”* (Diana, F28)

*“They tell you to start with 20, then 40, then 80, and if that doesn’t work, they increase it. Damn, seriously? I saw my father with depression, bedridden for over a year, completely drugged up, they just left him there, knocked out.”* (Hans,M25)

In general, participants criticized the way treatment dominated clinical consultations, often reducing them to a process of prescription rather than dialogue:

*“What’s wrong? Oh, you’re doing so badly, so badly. Here, take this, this, and this. I mean, you see I’m crying and you already know what pill I should take? Fine.”* (Diana, F28)

*“Hello, good afternoon, how are you? Here you go-bam, bam, bam-take your prescription and move along* [...] *The psychiatrist just asks, ‘How are you? How’s the medication going? Keep fighting, and take this.’”* (Carlos, M47)

### The effects: The pharmakon

*-”And with lorazepam, don’t you fall asleep during the day?”* (Beatriz, F35)

*-”No.”(Hans, M45)*

*-”Wow, it must be me...”* (Beatriz, F35)

Almost all patients acknowledge favorable effects from the medication, such as feeling calmer, sleeping better, and overthinking less. However, there is a nearly universal belief that medications are a “help” but do not cure:

*“It’s not that they make me happy, but I am better.”* (Beatriz, F35)

*“I don’t really notice a great improvement.”* (Diana, F28)

*“They don’t bring joy, but they calm you down.”* (Pedro, M58)

*“It’s like when you have a headache, it helps, but it doesn’t take it away.”* (Carlos, M47)

Lucas, although one of the least critical of psychotropic drugs, expresses that he uses them to *“be able to function, go outside.”* Eduardo (H26) describes how the calming effect of the medication allows him, even if he doesn’t go out, to have a clear enough mind to play on the console or computer. As Hans puts it:

*“With the medication, yes, you seem to feel better, but not in a tremendous way - just enough to survive.”* (Hans, M45)

The most euphoric sensation is only felt during the first two or three weeks of use and then fades away. The main effect described for antidepressants is feeling more motivated to do things, an impulse to act, rather than a sensation of happiness. For anxiolytics, it is being able to calm down a bit and sleep better.

Most patients report not feeling a major difference in their personality, though some mention that taking psychotropics makes them return to how they used to be:

*“It changes me, but it brings me back to who I was; I feel much more like the time when I wasn’t suffering.”* (Lola, F55)

Others do perceive that they stop being themselves:

*“I don’t know, but I feel like it doesn’t let you be yourself. It turns you into something you’re not, even if just during the time you’re medicated. Something that turns you into something you’re not...”* (Eduardo, M26)

Although this is more of a fear than an actual change:

*“You’re left thinking, today I feel this way, but am I feeling this way because I feel this way, or because of the medication, or what? At some point, I have the feeling of losing my benchmarks, losing the reference point of who I am and where I was.”* (Diana, F28)

Control (or its absence) frequently appears in patients’ narratives:

*“Since I’ve been on pills, I can control the outbreaks a little, but it’s like it stays inside.”* (Concepción, F55)

Although medications are seen as a means to control emotions, there is also the sensation of not being able to do so on one’s own. Marta (F21) explains during the consultation that she needs a job, is doing an internship, and feels overwhelmed because she wants to stop depending on her parents. She wants to control her anxiety on her own, without taking pills. Carlos (M47) attends his first psychology consultation after a period of psychiatric treatment because he learned that psychological therapies exist, and he wants to control his emotions and feelings, remove his anxiety, and stop the medication. He would like to be a little freer: *“to be myself and control.”*

Diana (F28) started with reactive depression due to the stress and high competitiveness of her job as an erotic teleoperator. In the mutual support group, she explains everything she takes: *“Zymbalta, Orfidal, Deprax, Enoltril, and recently they gave me another one, abilin or something like that, it’s an antipsychotic that made things even worse.”* Another participant in the group, Beatriz, asks if with so many pills she feels like she did before:

-*“I don’t think you act or are as alert.”* (Beatriz, F35)

-*“Sometimes I feel really strange, like very distant.”* (Diana, F28)

When she speaks, her discourse is not in the first person. When asked something during group sessions - she almost never speaks unless someone directly addresses her - she almost always responds by saying that her partner or some family member has suggested something similar, but it’s always something suggested from the outside. The combination of five psychotropic medications has not made her feel better than a year ago when I interviewed her; in fact, it has made her feel worse. The psychiatrist’s response in each session is to increase the dosage or add a new medication. Months later, when she interrupts the treatment and returns to the group, her discourse has changed: she is much more lucid and aware of what is happening to her.

If, alongside control, the idea of disconnection arises, and the notion that “things don’t affect me as much,” this disaffection is also perceived by some participants as strange and foreign, in the sense of not being oneself:

*“The first time they gave me Esertia, at first I didn’t feel anything, but then little by little, things affected me less, both good and bad.”* (Diana, F28)

[When stopping the medication] *“I feel like, wow, I’m back to being myself. Because the pills limit you so much, you lose sensitivity to situations, you become a bit robotic. If a movie made me cry my eyes out, with the pills, nothing, or on the other hand, I can’t even laugh out loud. It leaves me more frozen. I feel like it passes... like neither here nor there.”* (Concepción, F55)

The loss or decrease in libido, the inability to reach orgasm or ejaculate, is a common issue in consultations with psychotropic drug users. The alternative they often encounter is adding more medication, sometimes with interactions or new side effects, so they often end up accepting the absence of sexuality, which is discussed in the group as another aspect of the deterioration of the relationship.

Other side effects may include “*pancreatitis*,” “*weight gain, you look bad physically*,” “*dizziness as if I’m going to faint*,” or “*my legs don’t hold me up*.” It is also mentioned that sometimes they drop things, experience colic in the liver or kidneys, have nightmares, or “*are confused with a junkie*” because of how they move due to the medication. One of the most frequent complaints is feeling “*very sluggish*,” “*like a zombie*.” This figure is repeated by several interviewees: *“You walk like a balloon, like a zombie”; “like a zombie, like you’re doped up.”* This expression condenses two elements we’ve discussed: the fear of the absence of control and the feeling of not being fully alive or that things don’t affect you.

The effects differ from person to person. The psychiatrist in the consultation explains it to the patients this way: *“In psychiatry, we’re sort of blind. We have to try because what works for one person doesn’t work for another.”* In this sense, in the group, participants expect to have the same effects from the medications, only to discover that these vary from person to person. Doubts also arise about what is the effect of the medication and what is a symptom of the disorder. Complaints are constant, though the professional often doesn’t give them much attention; they are simply reclassified in the report as “ruminations about the treatment,” turning them into another symptom of the illness.

*“The last time was a bit strange: it seems like you don’t want to take the pills. I don’t want to take them, but I will take them if necessary. But I don’t know, I mainly told her about the side effects and that they weren’t agreeing with me. And she changed a bit: ‘Well, I’ll change it, but I’m not convinced that it’s really not agreeing with you; I think you believe it’s not agreeing with you, and that’s why it feels bad.’”* (Diana, F28)

The participants themselves have similar doubts:

-*“What happens to me is that I don’t have much memory, I’ve lost a lot, I forget things, and my concentration is terrible, and I have problems because then I don’t even remember what I said. Is it because of the treatment or the depression? I don’t know.”* (Beatriz, F35)

-*“Yes, I noticed during the times when I was taking the medication, studying, retaining things-wow, for me at least, it was hard. They told me it was all in my head, [laughs], yeah, yeah, whatever, no way.”* (Hans, M45)

-*“And could that be because of the depression, forgetting everything and concentration, or is it because everyone has their head like that, like distracted?”* (Beatriz, F35)

-*“I noticed it a lot, it caused me anxiety to forget.”* (Hans, M45)

-*“It’s starting to cause me that.”* (Beatriz, F35)

*-“It generated like dissatisfaction and frustration to spend hours and hours studying and not getting results. It even depressed me... You’d get caught in a kind of bubble, and in the first stage, that kind of drowsiness was interesting because it made you forget your problems. But then there comes a moment when you want to get back in the game, you don’t want to stay in the bubble.”* (Hans, M35)

### The consultation as negotiation

In psychiatric consultations, symptoms - mostly physiological signs - are reviewed to adjust the medication. The level of activation, sleep, mood, appetite, and nervousness are assessed, along with any potential side effects of the medication, such as vision problems, libido issues, dizziness, headaches, or other effects controlled through tests that provide data on liver function or cholesterol levels. Based on multiple variables, the dosage can be increased or decreased, the timing of the medication can be adjusted, pills may be added to counteract side effects, or the medication itself may be changed, always with the possibility of losing the benefits of the current one. This dynamic involves continuous decisions and trade-offs regarding medication in a cost-benefit balancing act.

It is common to try different medications until finding the one that works best. Carlos (M45) went through four medications: the first caused dizziness, the second made him “*too high*,” the third gave him severe stomach pain, and the last one caused facial paralysis. Some people give up, while others endure.

Consumers of psychotropic medications play an active role in the decision-making process regarding their medications. It is often they who propose stopping or reducing the dosage, with or without supervision, although not all professionals are equally flexible. In some cases, this involves a negotiation:

*“I say, I’ve reached my limit, I think I’m strong. They don’t fully trust me, they don’t take me off them, but they start reducing the dosage* […] *Before going to the doctor, I had already reduced half, I arrived without any. And the doctor said: ‘Well, if you’ve already stopped, then nothing.’”* (Concepción, F55)

It is also common that not only the decision but the withdrawal itself begins before the consultation and medical supervision. Several patients mention behaviors like Diana’s:

*“I had been on it for a year and a half, and they kept increasing it, and increasing it, and increasing it. We started at 5 mg, and by now I was at 20. After a year and a half, I wasn’t seeing any progress, and if I’m not making progress, I don’t want to keep taking these pills. Yes, I’m really scared of getting hooked. You read the leaflets and think: ‘My God, what are they giving me?’ I changed houses, I was more upbeat, and after two days, I had forgotten about it, and I stopped.”* (Diana, F28)

Medication becomes synonymous with discomfort, both its quality and quantity, so it is common to hear in consultations, in response to the question about how a patient feels: *“I’ve been on Orfidal for a month, today I’ve already taken three.”* Taking medication is associated with being sick, and stopping it is linked with being well. Clara (F26) speaks of herself as sick during the time she was medicated, but now, as she manages to control herself without medication, she no longer sees herself that way.

### Autonomy, dependency, and chronicity

*“A chronic disorder is a big business.”* (Clara, F26)

The main concern for participants regarding medications is the dependency they generate. Hans explains it this way:

*“Self-esteem grows if you can control it without needing to take anything because you think I’m capable. The idea is, okay, I have these shortcomings - everyone has certain shortcomings in one way or another - damn it, I’m capable of solving the problem through work. I don’t need to turn to chemistry, which also has side effects, for these kinds of processes.”* (Hans, M45)

The value placed on the use of both legal and illegal drugs also reflects a negative view of substance dependency. Many participants report feeling “like a junkie.” Patricia explains why she dislikes medication: “It feels like a drug, a real drug, a dependency drug”; or Carlos (M47): *“Oh, I’m missing my little pill... You have two problems: anxiety and addiction.”* Several participants report experiencing physical symptoms when discontinuing medication, with withdrawal syndrome often described as similar to that of other drugs:

*“I felt a physical response from stopping it. I started shaking, went to the doctor, I was walking like I was high, with dilated pupils.”* (Clara, F26)

Almost all participants live with fear and concern about the moment of stopping medication, and some have already accepted that they may never stop taking it. When someone feels better, they do not know whether, after stopping the medication, they will return to the same point that led them to begin treatment, meaning there is no certainty of real improvement in their distress:

*“Yes, the pills work, but let’s see, the real test is when I don’t take them. It’s a con job, the pill doesn’t cure me, I’m not diabetic. The pill slows me down, it lets me survive with a little dignity, but if when I stop them I fall back into the loop, then what good have they done me? They haven’t done anything.”* (Concepción, F55)

Many participants improve at the start of treatment but begin to feel worse a few months after discontinuing the medication. Relapses are not only frequent, but *“worse”* because of the *“loss of trust”* they cause. In line with the idea of being capable and doing it on one’s own, Beatriz views not being able to stop the pills as a sign of her weakness:

*“I’m clear about it because I’ve tried twice and no... I want to stop because of the side effects of all the pills, and because my mom is on my case, telling me I have to be strong, that I have to get through it myself. Because she also had some signs of depression when she separated, and she could do it on her own. But we’re not all the same, mom, if we were all the same, there wouldn’t be pills. Some people are weaker, and others are stronger. I’ve tried to stop, but after two or three months, I relapsed again.”* (Beatriz, F35)

There is hardly any information from professionals about treatment expectations or the possible course of the illness. It is recommended to continue treatment for a while despite improvements, without clarifying whether remission will occur. Psychiatrists often downplay the importance of taking medication, as Lola, who is diagnosed with reactive disorder due to a difficult situation at her workplace, explains:

*“It worries me, I don’t like it, but then when I talk to them [the psychologist and psychiatrist] and tell them I don’t like taking medication, they say it’s nothing, that it’s improving my quality of life at work, so I guess the body also deteriorates without taking it.”* (Lola, F55)

The daily lives of people in the mental health center are marked by repeated attempts to reduce or eliminate medication:

“Here I am still fighting to see if they can lower my dose and take me off it, but as soon as I stop taking something, I get really nervous.” (Blanca, F49)

### What’s happening to me? The attribution of distress

In the accounts of the interviewed individuals, the issue of the brain barely appears. Neither during observation nor spontaneously in interviews is it expressed as an explanation for their suffering. In the observation, only once did the wife of a patient ask the psychologist how they knew that her husband was lacking a vitamin in his brain when no tests had been done. The husband indicated that it was serotonin. This is the only variable that emerges in the group when discussing the possibility of depression having a biological cause:

*“The serotonin issue, it’s about synapses, they say, it seems to be studied, the reuptake, that releasing more serotonin is fostered by the pills. Depressed people, I guess not all of them, but some biologically reuptake too much serotonin, that seems to be...”* (Hans, M45)

Despite most people taking medication and noticing improvement, this does not automatically lead to attributing their distress as something biological. Only Hans, who is somewhat more familiar with the functioning of antidepressants, suggests this relationship:

*“If it’s not biological, why are we taking the serotonin stuff? It would be logical to think that we reuptake a lot of serotonin, it stays there in the synapse, and for others it flows like nothing, and they’re so happy, it seems that’s how it is with the pills.”* (Hans, M45)

However, despite recognizing the importance of social issues in the onset of distress, when discussing their particular experiences, participants end up referring to something intrinsic to them, some kind of personal flaw or defect. While social or labor-related factors appear repeatedly in their narratives, they are framed as secondary factors that exacerbate their chronic vulnerability. When talking about acquaintances or friends, the attribution is different. However, when crafting a personal narrative, the explanation always comes with personal limitations, which are seen as determining factors. Pedro, a unionist with serious work-related issues, firmly stated the first time I spoke with him: *“What’s clear is that mine was a reactive process.”* However, when discussing the chronicity of his distress in the group, he shared:

*“Maybe I already had a little something, my mother was depressive, I’ve improved in these six years, but I have times when I relapse… Some people are touched, or we are touched, we are more prone to this not disappearing completely, but I think in my case, I have some kind of factory defect.”* (Pedro, M58)

Similarly, Lucas, dealing with work-related stress, points to that responsibility for what happens to them:

“*I think I’m predisposed to have anxiety, that’s how I live life. Because I’ve had it inside me, I’ve forged this personality*.” (Lucas, M47)

Successive relapses are seen as a synonym for individual failure, thus reinforcing the sick role. Pedro, 58 years old, joined Comisiones Obreras (the Workers’ Commissions Union) at the age of 18 and dedicated his entire life to union struggles. After losing a lawsuit with his company six years ago - where the union didn’t even show up - he attempted suicide and was granted permanent work incapacity due to major depression. His image as a unionist gave way to that of an incapable worker due to illness.

When analyzing in depth the individuals interviewed who pointed to work as the main source of their distress, most displayed resistance behaviors, active responses to situations they considered unjust. Of the eleven interviewed who raised this issue, only two did not mention explicit resistance behaviors. That is, their suffering did not arise from accepting work conditions, but from rejecting them and from the conflicts this rejection caused with superiors. Several participants only took medication during their workday and stopped when they rested on weekends and during vacations. The main reason for taking medication was to withstand the pressure, bad attitudes, or reprisals. The following psychiatric record summarizes the daily routine of patients with work-related problems:

*“She attends the consultation with anxiety-depressive symptoms due to conflicts at work in 2010, feeling overwhelmed, with anxiety and multiple somatic symptoms,* [...] *as well as ruminations about the work problem. Antidepressant treatment was reinstated with progressive improvement of symptoms and favorable evolution, also becoming more capable of distancing from the problems that persist in the work environment.”* (Field Diary)

In the consultation, the psychiatrist’s words of encouragement reveal the connection between intervention and acceptance: *“The situation is the same, but you are better.”* Besides enduring unjust situations, many participants internalize guilt:

*“You don’t show up and say, hello, I’m Clara and I have stress problems, if you pressure me too much. But if you are already a person who suffers and they treat you like that...”* (Clara, M26)

## DISCUSSION

The subjectivation analyzed in our study occurs more as a problematization than as automatic internalization. In line with the analyses of Foucault’s later work, which questions the non-deterministic ways people relate to what he calls technologies of the self,^43^^,^^44^ the approach to the medical encounter happens as a negotiation, with an ambiguous reception. This ambivalence calls into question the view of medicalization as a process eagerly embraced by individuals and reveals the discomfort that the consumption of medication itself entails.

We can understand that the two logics present in the intervention (cerebral and therapeutic) operate simultaneously because the type of *self* that both propose corresponds to neoliberal subjectivity, that is, to the way we are mobilized in our society. It is about the ideal of an autonomous person, with initiative, who unconditionally accepts themselves without needing approval and who controls negative emotions, manages risks, and evaluates their mood, emotions, and physiological reactions.^45^ The neoliberal logic involves promoting competitiveness in companies but also inciting the individual to become a company themselves.^46^ In the subjectivity revealed by the participants’ narratives, aspects such as the fear of relapse and side effects become central, with an ideal of autonomy that is hard to achieve when one feels that change is not a result of personal effort and carries the feeling of responsibility for what happens to oneself.

### The pharmakon and the consultation as negotiation: Self-care and individual responsibility

The main feature highlighted by participants is the ambivalent relationship they establish with the medications they consume. Martin^47^ refers to this quality with the ancient Greek term pharmakon, which simultaneously means remedy and poison. Medications, while partially alleviating individuals’ distress, generate new problems such as side effects and dependence, creating a new form of discomfort.^21^ In contrast to the idea of the possibility of improvement or designing a self at the consumer’s will, the participants in the study do not seek to be happy or forget their problems, nor do they wish to become different people. They speak of survival and returning to the productive cycle, even at minimal levels and at the cost of numerous sacrifices. Among these sacrifices are the capacity to be affected by things, the concentration to study, or sexual desire. When these issues are presented in the consultation, they are often minimized or even reinterpreted as *“ruminations about the treatment,”* turning them into just another symptom of the illness. But the reality is that it is impossible to separate the side effects from the experience of medication.^31^

Consumers of psychotropic drugs constantly weigh and make decisions about their medication. While the biomedical approach typically criticizes self-medication, this is consistent with the waiting times between appointments, the frequent on-demand prescriptions (such as anxiolytics), and the scrutiny of symptoms and reactions that takes place in the consultation. While healthcare professionals tend to view self-medication as negative, practices that lead in that direction are continuously promoted within the sector.^48^

According to Rose,^5^ the logic of personal development and authenticity within therapeutic culture also exists in pharmacological intervention, as medications are prescribed and consumed as a way of taking control over oneself. That is, they would not contradict the technologies of the self described by Foucault,^49^ as the ways individuals experience, understand, judge, and conduct themselves. Medications would also be technologies for conducting the relationship with oneself, in the form of autonomy through the active role of the consumers. The ideal of control frequently appears in patients’ narratives, though always as something to be achieved, tied to autonomy. Although medications are seen as a means to increase control over emotional reactions, they coexist with the sense of being unable to do so on one’s own, with the perception of being unable to manage emotions autonomously,^50^ and with one of the most common effects described by consumers in this and other studies: feeling like a zombie, synonymous with lacking consciousness.^51^

The first characteristic of pharmaceutical subjectivity found in participants would then be the establishment of self-governance through self-care. The management of the *pharmakon* - the dual nature of medications as both beneficial and harmful - involves constant monitoring and decision-making in the form of a cost-benefit balance.

### Chronicity and vulnerability

The primary concern of the participants is the dependence generated by medications. There is a contradiction between the ideal of autonomy that characterizes neoliberal subjectivity, pharmacological dependence, and the feeling of not achieving improvement on one’s own. The repeated attempts by participants to lower or eliminate their medication without success result in the chronicity of the discomfort in a significant number of people, instead of a cure. The most visible case is that of sleeping pills, which paradoxically end up causing insomnia,^52^ but antidepressants have a similar effect.^53^^,^^54^ This suggests a model of illness in which the individual who recovers is at risk of future relapses, and medication is prescribed both for the risk and for the condition. The psychotropic drug consumers interviewed view themselves as sick, at risk, and dependent. The recurring relapses are understood as synonymous with individual failure.

The fact that the intervention revolves around medication has partly resulted in the discomfort being perceived, interpreted, and lived as a medical ailment, often independently of its intensity or origin. Charmaz^55^ describes, as one of the main causes of suffering in people with chronic illnesses, the loss of the self. Old images disappear, and suffering becomes the center of life. The image of many participants in our study is that of patients, unable to work, tolerate stress, etc. A new self marked by vulnerability, failure, and risk. Martínez-Granados et al.^56^ also found that patients with chronic mental health diagnoses saw medication as a symbol of vulnerability and fragility. Moreover, this vulnerability is accentuated in the processes of withdrawal and/or reduction of medication.

The ideals of autonomy, work, and individual responsibility appear as the reverse of the vulnerable *self* in the role of the sick person. In a society that prioritizes doing over being, the inability to carry out conventional tasks makes it difficult to maintain a meaningful life.^55^ Conceiving our biography in terms of illness leads to a permanent reflexivity in which our selves are continuously problematized and pathologized. Life becomes a series of constant revisions and improvements.

The second characteristic of pharmaceutical subjectivity found in our study is the chronicity of the treatment, related on the one hand to the dependence generated by medications and, on the other, to the notion of risk around which the treatment is articulated. The feeling of being at constant risk - of relapse or worsening - is another trait of pharmaceutical subjectivity.

### Reification of distress

In the context of the study, the intervention in the psychiatric consultation is limited to the prescription of psychotropic drugs. The relationship that the people studied establish with the medications is complex and ambivalent, but in general, they emphasize that prescription is overused in comparison to therapy. The treatment guides the entire intervention, from diagnosis to the clinical encounter itself. The centrality of prescription shapes the imagination surrounding the attributions of distress and the possibilities that open up when it comes to addressing it. Although the explanation for the suffering goes beyond the biological, the practice ultimately determines its approach, which in the consultation appears as a matter of physiological self-regulation between symptoms and side effects. As Martínez^57^^)^ describes, if I modify my brain, there is no need to modify the world, so I end up prioritizing the self over the social world.

Unlike studies in which psychotropic drug consumers locate the origin of their distress in the brain,^25^^,^^58^ in our context, brain narratives barely appear, not even coexisting with others, as found in other analyses in our country.^59^ The public health system limits commercialization and marketing in Spain, which targets professionals rather than patients, unlike the direct-to-consumer ads in the US.^37^ Cultural, social, and contextual factors then determine the imagination built around the use of medications, diagnoses, and mental distress.

However, when participants speak of their particular experiences, they always end up referring to something that is inherent to them, to some kind of flaw or personal deficiency. The more social explanation is short-circuited by the biomedical logic through the generation of the sick role. Suffering is not encoded exclusively in biological terms, but it is articulated around the figure of vulnerability, weakness, and deficit. Personal anguish is framed as a problem to be treated with pharmaceutical solutions, aligning with the neoliberal imperatives of self-optimization, rather than addressing the structural or social causes of distress.

Furthermore, the distress often forms isolated forms of resistance to living conditions, for example, in work. And these resistances or challenges, translated into biomedical language, are codified as individual flaws, decontextualized and emptied of their symbolic, singular, and social content. The intervention, in these cases, appears as a form of reification in that it intervenes on subjectivities that have deviated from the normative.

The third characteristic found would then be reification, which involves the establishment of an explanatory model of distress that limits social factors to mere aggravators, placing at the center of the justification for suffering an individual predisposition, a form of deficit or flaw for which the person is less able to cope with difficulties.

### Limitations and current context

It is important to emphasize that by pointing out the negative effects that psychotropic drugs can have, the intention is not to demonize the individuals who take them or the potential benefits they may have.^60^ Furthermore, despite the value of the narratives of psychotropic drug consumers - in the specific context of a public mental health center in Madrid during the previous economic crisis - the main limitation of the study is precisely its contextual scope. On one hand, the crisis we are experiencing now is different, with lower unemployment rates, and with a series of years in which improvements in macroeconomic figures do not go hand in hand with improvements in living conditions. This suggests that the sense of individual responsibility for failure may be less pronounced in the current context. On the other hand, despite recent studies in the U.S. continuing to find an increase in the tendency to frame suffering in neurobiological terms rather than external life events,^61^ it would be necessary to assess how the pandemic may have impacted narratives around suffering, bringing the effects of context on mental health to the forefront. Therefore, it would be important to continue questioning and listening to the voices of individuals who consume psychotropic drugs within the new context we now inhabit.

## CONCLUSION

The psychiatric intervention in the context of this study implies a certain one-directionality in the prescription process and the relationship with the professional. Not only is there no neurobiological explanation in the participants, but there is also no clear demand for psychotropic drugs, which are generally seen as a lesser evil to *“survive”* in the absence of the possibility to undergo therapy. The intervention produces a form of subjectivity, which we can call “pharmaceutical,” characterized by its ambivalence and the need to negotiate continuous adjustments to minimize side effects and avoid falling into chronicity.

Key elements of this subjectivity include reification, where the feeling of being in deficit takes precedence; self-governance, which involves constant monitoring that accentuates the sick role; and chronicity, which involves perceiving oneself as always at risk and therefore vulnerable. Against the notion of medicalization, which presents a one-directional and simplistic process, the term “pharmaceutical subjectivity” has been preferred to account for the complexity of the effects of the intervention. In the resulting subjectivity, aspects such as fear of relapse and side effects, autonomy (which is difficult to achieve when one feels that change is not a result of personal achievements), and the responsibility for suffering and self-governance - central elements of neoliberal subjectivity - become crucial.
